# Neonatal Pain-Related Stress and *NFKBIA* Genotype Are Associated with Altered Cortisol Levels in Preterm Boys at School Age

**DOI:** 10.1371/journal.pone.0073926

**Published:** 2013-09-16

**Authors:** Ruth E. Grunau, Ivan L. Cepeda, Cecil M. Y. Chau, Susanne Brummelte, Joanne Weinberg, Pascal M. Lavoie, Mihoko Ladd, Aaron F. Hirschfeld, Evan Russell, Gideon Koren, Stan Van Uum, Rollin Brant, Stuart E. Turvey

**Affiliations:** 1 Department of Pediatrics, University of British Columbia, Vancouver, BC, Canada; 2 Developmental Neurosciences and Child Health, Child & Family Research Institute, Vancouver, BC, Canada; 3 Molecular Toxicology, University of Western Ontario, London, ON, Canada; 4 Cellular and Physiological Sciences, University of British Columbia, Vancouver, BC, Canada; 5 Statistics, University of British Columbia, Vancouver, BC, Canada; Boston Children’s Hospital and Harvard Medical School, United States of America

## Abstract

Neonatal pain-related stress is associated with elevated salivary cortisol levels to age 18 months in children born very preterm, compared to full-term, suggesting early programming effects. Importantly, interactions between immune/inflammatory and neuroendocrine systems may underlie programming effects. We examined whether cortisol changes persist to school age, and if common genetic variants in the promoter region of the *NFKBIA* gene involved in regulation of immune and inflammatory responses, modify the association between early experience and later life stress as indexed by hair cortisol levels, which provide an integrated index of endogenous HPA axis activity. Cortisol was assayed in hair samples from 128 children (83 born preterm ≤32 weeks gestation and 45 born full-term) without major sensory, motor or cognitive impairments at age 7 years. We found that hair cortisol levels were lower in preterm compared to term-born children. Downregulation of the HPA axis in preterm children without major impairment, seen years after neonatal stress terminated, suggests persistent alteration of stress system programming. Importantly, the etiology was gender-specific such that in preterm boys but not girls, specifically those with the minor allele for *NFKBIA* rs2233409, lower hair cortisol was associated with greater neonatal pain (number of skin-breaking procedures from birth to term), independent of medical confounders. Moreover, the minor allele (CT or TT) of *NFKBIA* rs2233409 was associated with higher secretion of inflammatory cytokines, supporting the hypothesis that neonatal pain-related stress may act as a proinflammatory stimulus that induces long-term immune cell activation. These findings are the first evidence that a long-term association between early pain-related stress and cortisol may be mediated by a genetic variants that regulate the activity of NF-κB, suggesting possible involvement of stress/inflammatory mechanisms in HPA programming in boys born very preterm.

## Introduction

It is well established that the hypothalamic-pituitary-adrenal (HPA) axis or stress system is particularly sensitive to programming by early life events [1]–[8]. Dysregulation of immune/inflammatory responses may play a central role in mediating early programming effects [9]. Studies in the field of early programming have focused primarily on heightened stress reactivity later in life, often measured in terms of HPA responses to acute stressors. In contrast little is known about effects of programming on endogenous stress levels long after a period of early stress has terminated.

Infants born very preterm (≤32 weeks gestation) are exposed to considerable procedural pain-related stress during weeks to months of life-saving procedures during hospitalization in the neonatal intensive care unit (NICU), that appears to impact the HPA axis long after NICU discharge [8], [10]. Importantly, the neuroendocrine system, the immune system and the central nervous system are linked in a complex regulatory network, with extensive bidirectional communication between and among these systems (e.g. [11], [9]). Glucocorticoid hormones can regulate expression of immunologically related genes [11], [12], and conversely, dysregulation of immune/inflammatory responses may play a central role in mediating early programming effects of the HPA axis [9]. The potential role of stress hormones in mediating immune/inflammatory function and in turn, the role of immune function/inflammation in mediating changes in the HPA axis in children born very preterm have not been addressed to our knowledge.

Cortisol levels are known to be relatively low while preterm infants are in the NICU [13]–[16]. Long after NICU discharge, the trajectory of cortisol activity over time appears to be altered [8]. In a longitudinal cohort, we found that greater cumulative neonatal pain-related stress (higher number of skin-breaking procedures from birth to term adjusted for neonatal medical confounders) was associated with altered baseline cortisol levels at 8 and 18 months corrected age compared to infants born full-term term [8], [10]. Importantly, endogenous cortisol levels play an important role in brain function [17]–[19] and in physiology and metabolism [20]. Thus alterations in HPA activity and responsiveness at school age in children born preterm have important functional implications.

Stress and adversity in early life can program a phenotype of exaggerated adrenocortical and inflammatory responses to challenge [21]. Environmental stress can enhance or suppress aspects of the immune response by altering activity of the cellular signaling pathways that regulate inflammation, including the proinflammatory transcription factor NF-κB [9]. Long-term alterations in HPA responsiveness that result from early life stress and adversity may also play a role in modulating immune function. Moreover, early life stress, including pain, shows sex-specific effects in rodents, however the direction varies depending on numerous factors such as age and type of exposure (e.g. [22]); therefore, we examined gender differences. Several studies have reported associations between maternal depression and altered child basal cortisol levels [23], therefore we examined maternal depression and anxiety as potential social confounders.

In the present study we first determined whether the increased cortisol levels observed in children at 8 and 18 months persist to school age in children born very preterm. Then to address the etiology of HPA alterations in these children, we examined whether neonatal inflammation may be involved in programming of the HPA axis by early life stress. Specifically, we investigated whether common genetic variants in the promoter region of the *NFKBIA* gene modulate the long-term associations of neonatal procedural pain-related stress with HPA axis programming in children born very preterm. The *NFKBIA* gene encodes IκBα, a critical negative regulator of the transcription factor NF-κB [24], [25]. NF-κB regulates the expression of the majority of proinflammatory cytokines, chemokines and leukocyte adhesion molecules, as well as pro-survival and anti-apoptosis genes; moreover, dysregulation of NF-κB is a known consequence of early life stress [26], [27]. Specifically, we assessed functional genetic variants in the promoter of *NFKBIA.* It has been established that individuals carrying the minor allelic variants at rs3138053 and rs2233409 have lower expression of both the *NFKBIA* gene expression and IκBα protein and this decrease in negative regulation is associated with higher Toll-like receptor-mediated inflammatory responses [28]. Importantly, in the present study, we used hair cortisol as an integrated measure of chronic stress that provides a well-validated index of endogenous HPA axis activity over time [29]–[31] in contrast to salivary or serum cortisol that provides an acute measure at a single point in time, varies with time of day, and reflects the more immediate stress context. Furthermore, in a subset of children born preterm that consented to provide a blood sample at age 7 years, we compared the levels of NF-κB-driven secretion of interleukin 6 (IL-6) and tumor-necrosis-factor alpha (TNFα) between subjects who varied in the promoter regions of the *NFKBIA* gene.

Cumulative neonatal pain-related stress was operationalized as the sum of all skin-breaking procedures, adjusted for medical confounders. The magnitude of response to a procedure in preterm neonates does not directly reflect the degree of invasiveness. Rather, sensitization to what has occurred in the past hour [32], past 24 hours [33], and cumulatively since birth [34], underlies reactivity. Sensitization is mediated at the spinal cord level, and is a phenomenon of the immature central nervous system that is excitable to incoming stimuli, inducing a wind-up phenomenon that affects reactivity to subsequent stimuli [35]. Therefore we use the number of skin-breaking procedures from birth to hospital discharge as an index of “pain-related stress” and did not adjust for extent of each procedure.

Our approach reflected the theoretical framework of long-term effects of major psychological childhood stress proposed by Miller, Chen and Parker [9], and aimed to extend their model to long term effects of physical stress in very immature preterm neonates. We hypothesized that at age 7 years: 1) hair cortisol levels will differ in children born very preterm compared to full-term, and differ by gender; 2) among the very preterm children, procedural pain-related stress in the NICU, quantified as number of skin-breaking procedures from birth to term equivalent (adjusted for medical and social confounders), will be associated with altered hair cortisol levels at age 7 years; and 3) genetic variation that impacts the regulation of NF-κB will interact with the degree of pain-related stress in the NICU and be reflected in hair cortisol levels at age 7 years. To our knowledge, this is the first study in very preterm children to address the etiology of HPA activity in relation to a transcription factor critical in stress and immune function, and the first study in this population of a cumulative index of stress long after hospital discharge.

## Materials and Methods

### Ethics Statement

The study was approved by the Clinical Research Ethics Board of the University of British Columbia and the Research Ethics Board of the Children’s & Women’s Health Centre of BC, and conforms to the conventions set out in the Declaration of Helsinki.

### Participants

**Figure 1:**
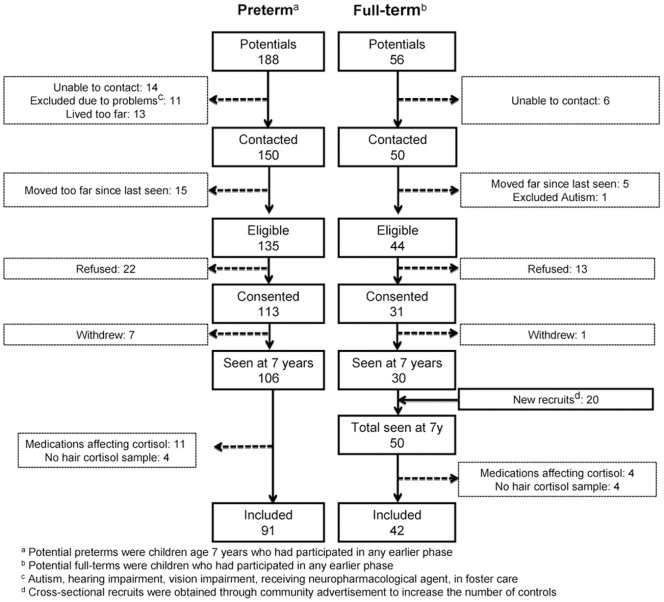
Recruitment flow chart.

A total of 133 school age children, 91 born preterm (PT) at ≤32 wk gestational age (GA) (M = 42, F = 49; mean gestation 29.6 wk; SD = 2.4; mean age at testing 7.7 yrs; SD = 0.3) and 42 full-term (FT) healthy controls (M = 15, F = 27; mean gestation 39.9 wk; SD = 1.0; mean age 7.8 yr; SD = 0.8) were included in the present study as part of a longitudinal study on the long-term effects of pain-related stress in children born very preterm (e.g. [8], [36]) (see Table 1). Excluded were children with major sensory, motor, or cognitive impairment (Wechsler Intelligence Scale for Children - Fourth Edition (WISC-IV, full scale IQ <70). In addition, children currently on glucocorticoids or other medications that affect cortisol (e.g. to treat asthma or attention deficit hyperactivity disorder) were not included (11preterm, 4 full-term). A flow chart of recruitment is shown in Figure 1. Written informed parent consent and child assent was obtained. Hair samples were collected and psychometric assessment conducted on all the children who attended the study visit at age 7 years. We invited a subset of these children to undergo magnetic resonance imaging (MRI) at the B.C. Children’s Hospital, on a subsequent occasion. In order to measure cytokine levels, from the 91 preterm children who participated in this study, 54 (M = 23, F = 31) provided blood samples for cytokines assay, in conjunction with the visit to acquire MRI.

**Table 1:** Participant characteristics.

Characteristic	Preterm (n = 91)	Full-term (n = 42)	p value
**Child**			
Gestational age at birth (weeks) mean (SD)	29.6 (2.4)	39.9 (1.0)	0.0001
Birth weight (g) mean (SD)	1334 (431)	3500 (513)	0.0001
Child age (years) mean (SD)	7.7 (0.3)	7.8 (0.8)	0.239
Male gender, n (% male)	42 (46.2)	15 (35.7)	0.346
**Mother**			
Education (years) mean (SD)	15.8 (2.8)	18.6 (4.4)	0.0001
Beck Depression Index-II, mean (SD)	6.7 (6.1)	4.0 (4.7)	0.006
Trait Anxiety, mean (SD)	35.8 (8.5)	33.7 (8.3)	0.200
Married or Common Law, n (%)	80 (88)	40 (95)	0.128
Caucasian, n (%)	69 (76)	37 (88)	0.073
Asian, n (%)	18 (20)	4 (10)	
Other, n (%)	4 (4)	1 (2)	

### Neonatal Characteristics

Detailed systematic nursing and medical chart review was carried out by an experienced research nurse from birth to term equivalent age as described previously (e.g. [8]). Variables included, but were not limited to gestational age, birth weight, early illness severity (SNAP-II) on day 1, number of skin-breaking procedures (e.g. heel lance, intramuscular injection, intravenous or central line insertion), days of mechanical ventilation, postnatal infection confirmed on clinical laboratory testing, number of surgeries, and daily dose of morphine adjusted for daily weight. Cumulative neonatal pain-related stress was operationalized as the sum of all skin-breaking procedures, adjusted for medical confounders. Cumulative exposure to morphine was calculated as the average daily dose (i.e. intravenous dose plus intravenous-equivalent oral dose) adjusted for daily body weight, multiplied by the number of days of morphine administration. A summary of the neonatal characteristics of the preterm children is provided in Table 2.

**Table 2:** Neonatal characteristics of the children born preterm (≤32 weeks gestation).

Characteristic	Boys (n = 42)	Girls (n = 49)	p value
Gestational age (weeks) mean (SD)	29.5 (2.4)	29.8 (2.4)	0.612
Birth weight (g) mean (SD)	1374 (488)	1300 (376)	0.431
Neonatal pain-related stress^a^ mean (SD)	110 (86)	87 (75)	0.166
Cumulative morphine exposure^b^ (mean, SD)	1.3 (3.9)	1.3 (4.2)	0.995
Mechanical ventilation (days) mean (SD)	8.0 (11.8)	8.2 (16.9)	0.947
Illness severity (SNAP-II day 1) mean (SD)	11.6 (10.3)	9.4 (11.7)	0.365
Surgeries (number) mean (SD)	0.4 (0.7)	0.2 (0.6)	0.226
Postnatal infection (yes/no)	0.3 (0.4)	0.3 (0.4)	0.899
Mother’s education (years) mean (SD)	16 (3)	15 (3)	0.270
Beck Depression Index-II, mean (SD)	6.5 (6.2)	7.0 (6.1)	0.686
Trait anxiety, mean (SD)	33.5 (8.1)	37.7 (8.4)	0.018
Married or Common Law, n (%)	37 (88)	43 (88)	0.960
Caucasian, n (%)	29 (69)	40 (81)	0.162

a Number of skin-breaking procedures from birth to term, including each attempt (e.g. intubation, injection, line insertion, heel lance).

b Exposure to morphine (IV and PO combined after appropriate conversion) from birth to term was calculated as the average dose (mg/kg) per day adjusted for daily weight multiplied by the number of days on the drug.

### Hair Cortisol

Cortisol was assayed from hair as an integrated measure of HPA activity in the prior two months. Hair samples were collected from the vertex posterior of the head, as this area has been shown to have the lowest coefficient of variation in hair cortisol concentrations [37]. A cluster of hair strands (∼2–3 mm diameter) were cut at the base of the hair shaft from five small spots. The hair samples were secured on a cardboard card with tape, labeled, and stored in individual sealed plastic bags at room temperature until analysis. The most proximal 2 cm from the scalp of each hair sample was utilized for the assay. The hair sample was weighed, and 10–15 mg from each sample was placed in scintillation vials. To remove external contaminants, hair samples were washed twice by immersing each sample in 3 mL of isopropanol and incubating it at room temperature in a centrifuge at 100 RPM for 3 minutes. After decanting the isopropanol, samples were allowed to dry for at least 12 hours. After drying, 1 mL of methanol was added to each scintillation vial. Hair was then finely minced with surgical scissors, cleaning the scissors between samples. The vials were sealed with paraffin film and incubated for 16 hours at 50°C in a centrifuge at 100 RPM. Following methanol extraction each cortisol-containing methanol solution was transferred into 5 mL test tubes and evaporated on a test tube hot plate under a steady stream of nitrogen gas. The residue was then reconstituted with 250 µL of phosphate buffered saline. These reconstituted samples were analyzed using the salivary enzyme linked immunoassay kit (Alpco Diagnostics, Salem, NH, USA). The intra-assay and inter-day coefficients of variation were 8.9% and 5.1%, respectively. The kit reported a sensitivity of 1.0 ng/mL.

### Parent Questionnaires

Beck Inventory –2^nd^ Edition (BDI-II) [38]: is a 21-item self-report questionnaire widely used to assess the presence and severity of symptoms of depression in adults in both research and clinical settings. The BDI-II has an alpha coefficient of 0.80. State-Trait Anxiety Inventory (STAI Form Y-2; [39]: Trait (T-Anxiety) is a 20-statement self-report scale widely used to assess how the respondent generally feels. Trait anxiety, but not state anxiety was used, since hair cortisol is a cumulative index of longer-term stress.

### Genotyping

DNA was collected from each of the 91 preterm children and extracted from saliva using Oragene OG-500 and prepIT-L2P collection kit (DNA Genotek/OraSure Technologies Inc., Bethlehem, Pennsylvania). The *NFKBIA* (rs3138053, rs2233409) single nucleotide polymorphisms (SNPs) were genotyped using commercially available TaqMan SNP Genotyping Assays (C_73866_10, C_15945891_10) on the Applied Biosystems 7300 Real Time PCR System (Applied Biosystems, Carlsbad, California). SNPs were deemed acceptable for analysis if they had call rates >99% and frequencies did not deviate from Hardy-Weinberg Equilibrium (HWE) (p-value >0.05). Standard TaqMan protocols were followed as recommended by the manufacturer.

### Cytokine Assay

Samples were collected in 4 ml 75 USP unit sodium heparin tubes (BD Vacutainer®, Mississauga, ON), incubated at 36°C for 18–24 hrs with 0.01, 0.1, and 1 µg/mL of lipopolysaccharide (LPS) (Ultra pure E. coli LPS 0111:B4, InvivoGen, San Diego CA). The LPS-induced release levels of interleukin-6 (IL-6) and tumor necrosis factor alpha (TNFα) were then assessed on 60 µL of whole blood and 140 µL of RPMI media with LPS (total of 200 µL/well) using Enzyme-Linked Immuno-Sorbent Assay (ELISA) kits (Human IL-6 ELISA Ready-SET-go!®, and Human TNF alpha ELISA Ready-SET-go!®, eBioscience, San Diego CA). ELISA was performed according to the manufacturer’s manual. ELISA plates were read using SpectraMax® Plus384Absorbance Microplate Reader (Molecular Devices, Sunnyvale CA), and quantified by the SoftMax Pro software provided by Molecular Devices.

### Statistical Analysis

In preliminary analyses, t-tests were used to compare characteristics between the preterm and full-term groups. The *NFKBIA* rs3138053 and rs2233409 polymorphisms in our sample did not deviate from Hardy-Weinberg equilibrium (p = 0.55 and 0.49 respectively). Pearson chi-square test was then used to compare the gene allelic distribution between preterm girls and boys. To address the central study questions, univariate ANOVA was carried out on the hair cortisol level, with group and gender as between-subjects factors, with concurrent self-reported mother anxiety and depressive symptoms as covariates in ANCOVA. Generalized Linear Modeling (GZLM) was used, for the preterm children only, to examine cumulative neonatal pain adjusted for multiple clinical confounders, in relation to hair cortisol levels. R [40] was used for GZLM, using F statistics and the car package for type III tests. Confidence intervals were computed using R. To establish adequate power the confidence intervals should not include zero [41]. SPSS17 was used for the other statistical analyses. t-tests were used to compare area under the curve (AUC) of LPS-induced release levels of IL-6 and TNFα between CC and CT/TT variants of *NFKBIA* rs2233409 and AA and AG/GG of *NFKBIA* rs3138053 polymorphisms.

## Results

### Participant Characteristics

Characteristics of the preterm and full-term groups are provided in Table 1. As expected, the preterm group had significantly lower gestational age and weight at birth. The proportion of boys and girls in each group and child age at the 7 year visit did not differ between the groups. Mothers of children born preterm self-reported more symptoms of depression on the Beck questionnaire and fewer years of education. However both groups had relatively high educational background (preterms mean 15.8 years, and controls 18.3 years). Neonatal characteristics and genotype distribution between preterm girls and boys are shown in Table 2 and Table 3 respectively. There were no statistically significant differences in neonatal characteristics between boys and girls among the preterm children.

**Table 3:** Allelic distribution of *NFKBIA* SNPs between preterm boys and girls.

	Position relative to start codon	Genotype	Boys n = 42	Girls n = 49	p value^a^
*NFKBIA* rs2233409 g.4675C>T	−310 bp	CC CT TT	22 19 1	31 14 4	0.294
*NFKBIA* rs3138053g.4091A>G	−894 bp	AA AG GG	19 21 2	27 17 5	0.348

a Dominant model p-values from Pearson Chi-Square tests between boys and girls.

### Hair Cortisol, Group and Gender

**Figure 2:**
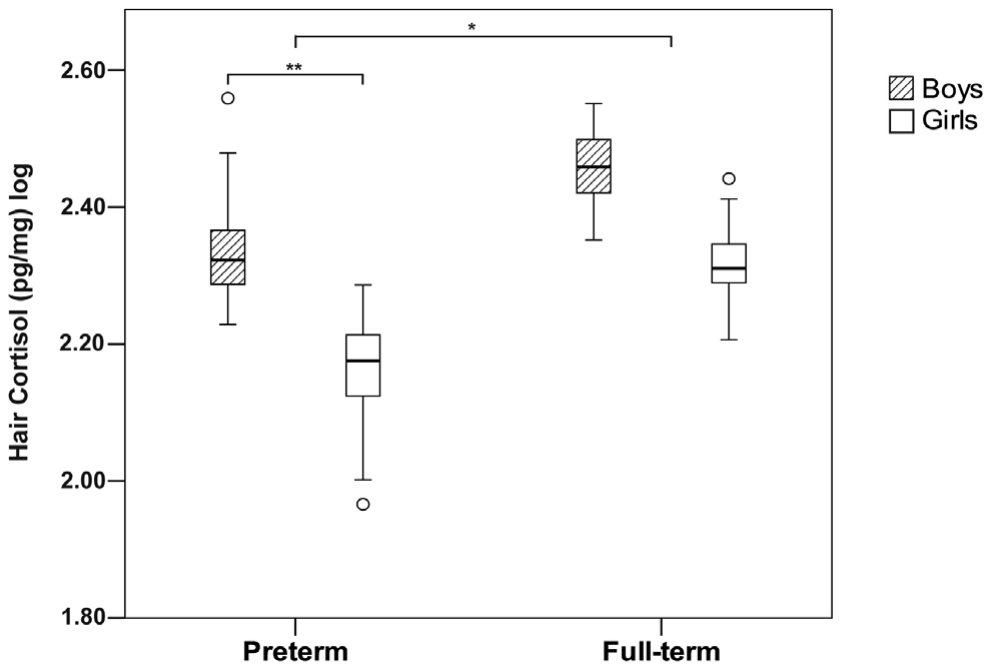
Hair cortisol level in boys and girls for the preterm and full-term groups adjusted for concurrent maternal depressive and anxiety symptoms and years of education. *p<.05, **p<.01.

Hair cortisol values were log transformed. Outliers >3 SD from the mean were winsorized such that the highest value within 3 SD was assigned [42]. On ANOVA, hair cortisol levels were lower in the preterm compared to the full-term children (p = 0.018), and lower in girls than in boys (p = 0.007). Subsequently, ANCOVA was used to control for concurrent maternal factors (self-reported depressive symptoms, trait anxiety, and years of education), and the main effects for group and gender remained the same (p = 0.006 and 0.007 respectively). There was a significance effect for maternal depressive symptoms (p = 0.041) but not maternal trait anxiety or number of years of education. The estimated marginal means for each group by gender are shown in Figure 2.

### Neonatal Pain, Morphine Exposure and Hair Cortisol in Preterm Boys and Girls

**Figure 3:**
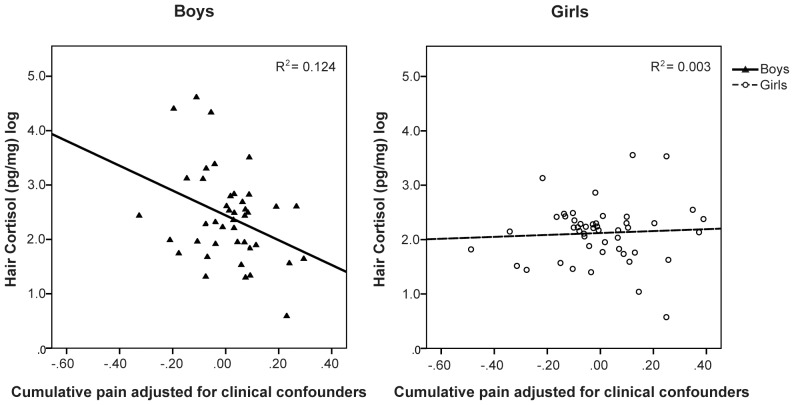
Relationship between neonatal pain-related stress adjusted for confounders (morphine exposure, gestational age at birth, illness severity SNAP-II day 1, postnatal infection, surgeries, days of mechanical ventilation) and hair cortisol at age 7 years in boys and girls born very preterm.

Since hair cortisol level differed by gender, we examined the relationship between neonatal factors and hair cortisol level separately for boys and girls. A GZLM model was constructed with number of skin-breaking procedures (cumulative pain from birth to term equivalent), number of surgeries, cumulative morphine (daily intravenous and oral doses calculated for equivalence, adjusted for daily weight), early illness severity (SNAP II on day 1), and days of mechanical ventilation as covariates, and postnatal infection as a factor, to predict hair cortisol level at age 7 years. In preterm boys, greater cumulative neonatal pain was associated with lower hair cortisol (B = −1.11, *p* = 0.037), independent of morphine exposure, early illness severity, days on mechanical ventilation, gestational age at birth, and postnatal infection, none of which were statistically significant. The GZLM parameters are shown in Table 4. There was no significant relationship between any of the neonatal predictors and hair cortisol level for girls (B = 0.085, *p* = 0.72). The association between cumulative neonatal pain and hair cortisol (adjusted for confounders) is shown for boys and girls in Figure 3.

**Table 4:** GZLM parameters for neonatal predictors of hair cortisol level at age 7 years in preterm boys.

			95% Confidence Interval			
	B^a^	Std. Error	Lower	Upper	t value	p value
Postnatal infection (yes/no)	0.38	0.21	−0.03	0.79	1.79	0.082
Pain-related stressb,c	−1.11	0.52	−2.12	−0.11	−2.17	0.037
Cumulative morphine dose^d^	−0.23	0.44	−1.09	0.63	−0.53	0.603
Mechanical ventilation (days)	0.01	0.01	−0.01	0.04	1.16	0.253
Gestational age (weeks)	−0.05	0.05	−0.15	0.05	−0.97	0.342
Severity of Illness (SNAP-II on day 1)	0.002	0.009	−0.02	0.02	0.24	0.814
Surgeries (number)	0.21	0.14	−0.05	0.48	1.57	0.126

a B is the unstandardized regression coefficient showing the effect on the outcome variable per unit change of each individual predictor.

b The confidence interval did not include 0, therefore power was adequate.

c Number of skin-breaking procedures from birth to term, including each attempt (e.g. intubation, injection, line insertion, heel lance).

d Exposure to morphine (IV and PO combined after appropriate conversion) from birth to term was calculated as the average dose (mg/kg) per day adjusted for daily weight multiplied by the number of days on the drug.

### Neonatal Pain, Hair Cortisol and *NFKBIA* Genotype in Preterm Boys and Girls

**Figure 4:**
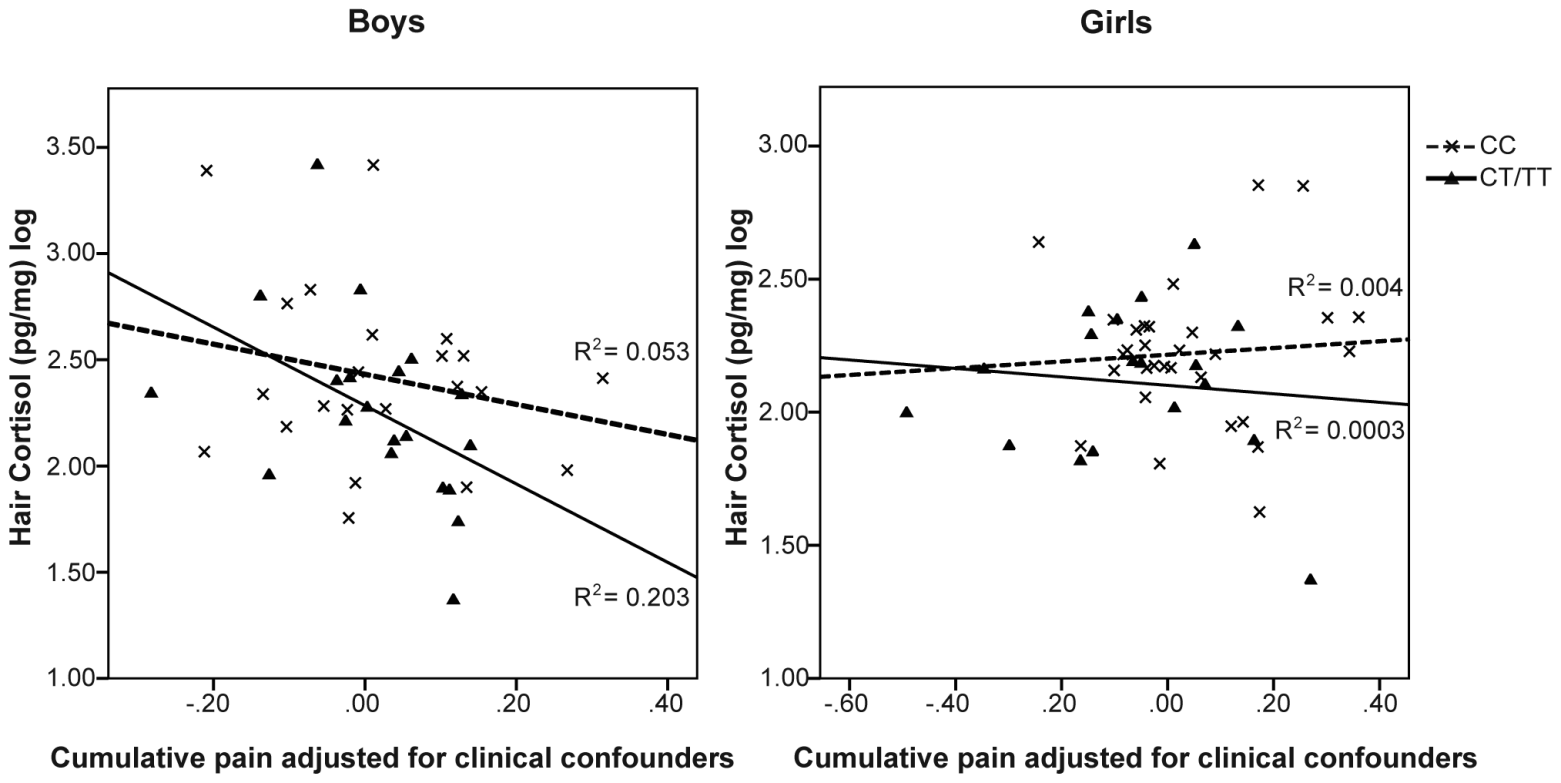
Genetic variants of *NFKBIA* rs2233409 and sex modulate the relationship between cumulative pain adjusted for clinical confounders and hair cortisol. Only in boys with a copy of the minor allele (CT/TT), higher cumulative pain predicts lower hair cortisol.

The allelic distribution for *NFKBIA* promoter SNPs rs2233409 and rs3138053 is provided in Table 3. For statistical analysis, children who were heterozygous and homozygous for the minor allele (CT or TT) were grouped together, and compared with children homozygous for the major allele (CC). The GZLM model was run again adding the *NFKBIA* polymorphism as a main factor in predicting hair cortisol level, and an interaction term (*NFKBIA* polymorphism X cumulative neonatal pain), in separate analyses for rs2233409 and rs3138053. In preterm boys, there was a significant relationship between hair cortisol and the interaction term of *NFKBIA* rs2233409 genotype and cumulative pain (B = −1.15, *p* = 0.03). For boys with a copy of the minor allele (CT or TT), lower hair cortisol was associated with greater neonatal procedural pain-related stress independent of morphine exposure, early illness severity, days on mechanical ventilation, gestational age, and postnatal infection. The GZLM parameters are shown in Table 5. For girls, there was no significant relationship between hair cortisol and the interaction term of *NFKBIA* rs2233409 genotype and cumulative pain (B = −1.12, *p* = 0.66). The interactions between the genetic variants and cumulative pain in relation to hair cortisol are shown in Figure 4. The GZLM was re-run for the *NFKBIA* rs3138053 genotype, with no statistically significant relationships for boys or girls. We then re-ran the models adding the concurrent stressors (maternal depressive and anxiety symptoms). Results remained the same for the interaction term *NFKBIA* rs2233409 genotype and cumulative pain for boys, *p* = 0.046; there was no statistically significant effect for the concurrent stressors for boys or girls. As before, there were no significant relationships with the *NFKBIA* rs3138053 genotype.

**Table 5:** GZLM parameters for neonatal predictors of hair cortisol level at age 7 years in preterm boys with *NFKBIA* rs2233409 in the model.

			95% Confidence Interval			
	B^a^	Std. Error	Lower	Upper	t value	p value
rs2233409 CC	0^b^	–			–	–
rs2233409 CT+TT^c^	2.10	0.10	0.15	4.06	2.106	0.043
Postnatal infection (yes)	0.28	0.21	−0.13	0.68	1.339	0.190
Postnatal infection (no)	0^b^	–	–	–	–	–
Pain-related stress^d^	−0.47	0.58	−1.61	0.66	0.813	0.422
Cumulative morphine dose^e^	−0.18	0.35	−0.86	0.51	0.503	0.618
Mechanical ventilation (days)	0.24	0.28	−0.30	0.78	0.882	0.384
Gestational age at birth (weeks)	−0.09	0.05	−0.02	0.02	1.624	0.114
Early Illness Severity (SNAP-II on day 1)	−0.002	0.009	−0.19	0.02	0.224	0.824
Surgeries (number)	0.16	0.14	−0.11	0.44	1.161	0.254
Interaction term: rs2233409 CC by pain-related stress	0^b^	–	–	–	–	–
Interaction term: rs2233409 CT+TT by pain-related stress^c^	−1.15	0.51	−2.15	−0.16	2.271	0.030

a B is the unstandardized regression coefficient showing the effect on the outcome variable per unit change of each individual predictor.

b Denotes parameter is redundant.

c The confidence interval did not include 0, therefore power was adequate.

d Number of skin-breaking procedures from birth to term, including each attempt (e.g. intubation, injection, line insertion, heel lance).

e Exposure to morphine (IV and PO combined after appropriate conversion) from birth to term was calculated as the average dose (mg/kg) per day adjusted for daily weight multiplied by the number of days on the drug.

### *NFKBIA* Regulated Cytokine Secretion in Preterm Boys and Girls

In preterm boys only, LPS-induced IL-6 and TNFα secretion from peripheral blood mononuclear cells was significantly higher in children with the CT/TT variant of *NFKBIA* rs2233409 compared to children with the CC variant. Likewise, children with the AG/GG variant of *NFKBIA* rs3138053 had significantly higher LPS-induced IL-6 and TNFα releasing levels compared to the AA variant (Table 6).

**Table 6:** LPS-induced IL-6 and TNFα releasing levels by *NFKBIA* SNP genotypes.

	rs2233409			rs3138053		
	CC (n = 11)	CT/TT (n = 12)		AA (n = 10)	AG/GG (n = 13)	
Cytokine	mean AUC* (SD)	mean AUC* (SD)	p value	mean AUC^a^ (SD)	mean AUC* (SD)	p value
**Boys**						
** IL-6**	3405.1 (2051.2)	5168.0 (1778.5)	0.039	3229.5 (2073.2)	5167.5 (1702.8)	0.022
** TNFα**	721.1 (573.7)	2116.3 (1798.2)	0.024	719.5 (604.7)	2010.1 (1763.7)	0.026
**Girls**						
** IL-6**	4419.1 (3411.3)	4962.0 (2264.5)	0.612	4702.9 (3220.4)	4604.2 (2540.0)	0.926
** TNFα**	1072.8 (1205.3)	1263.9 (982.4)	0.634	1156.4 (1205.6)	1156.4 (1009.9)	1.000

a mean area under the curve (10^−18^g^2^/ml^2^).

## Discussion

This study provides the first evidence, to our knowledge, that the HPA programming effects of procedural pain-induced stress in the neonatal period in children born very preterm persist to school-age. Furthermore, we utilized normal variation in the *NFKBIA* gene to examine whether genetic modulation of the inflammatory phenotype influences the effects of early stress. We found that for both boys and girls, hair cortisol levels as an integrated index of chronic stress, were lower in children born very preterm compared to full-term. However, the relationship between neonatal pain-related stress (adjusted for multiple confounders associated with prematurity) and hair cortisol was affected by presence of the minor allele for *NFKBIA* rs2233409 in boys, but not girls. These findings are the first to reveal that long-term effects of early pain-related stress exposure on the HPA axis are associated with normal genetic variation affecting *NFKBIA*/IκBα–the major negative regulator of NF-κB activity.

The present study extends our earlier work showing an altered trajectory of baseline salivary cortisol expression in very preterm-born infants from the same longitudinal cohort [8], [10]. These previous studies demonstrated upregulation of resting cortisol levels at 8 and 18 months corrected age in the the subset of preterm infants born at extremely low gestational age (ELGA, 23–28 weeks gestation). In the present study, we now found downregulation of hair cortisol overall in children born very preterm (both ELGA, 23–28 weeks and very low gestational age, VLGA, 28–32 weeks), in the same cohort at school age, suggesting persistent early programming. Cortisol levels in humans exposed to early adverse conditions can later show upregulation or downregulation depending on multiple factors including time since the stress occurred and the nature of the stress or trauma ([43], [44]. In particular, post-traumatic stress disorder is associated with a pattern of higher cortisol closer to the period of substantial stress, then lower cortisol later. Interestingly, *fetal* stress has been associated with low cortisol in adulthood, but only in a subset of individuals born low birthweight and post-mature (i.e. after 40 weeks gestation), whereas other subgroups of low birthweight adults displayed the opposite association between fetal stress and later cortisol concentrations [45]. Whether among infants born very preterm or growth-restricted at full-term, the lack of prospective longitudinal studies limits understanding of the etiology of early life stress and later variation in direction of cortisol. Our findings suggest such developmental trajectories long-term to be complex and likely gender-specific, requiring a lot of further research to elucidate the factors involved.

Relationships between dysregulation in cortisol levels and clinical manifestations are complex. A recent review of research on hair cortisol described contradictory findings on the direction of cumulative cortisol in relation to depression [46]. In general, long-term associations between HPA axis dysregulation and clinical manifestations, and the pathophysiological mechanisms underlying variation in direction of these relationships, are not well understood [47].

Our data are unique to our knowledge, as they arise from the only longitudinal cohort study of the developmental trajectory of HPA activity in children born very preterm compared to full-term, spanning infancy to school-age. In the only other study to compare cortisol levels in preterm versus full term children, high awakening salivary cortisol was reported in early adolescence in the preterm group [48], indicating that different information about HPA function may be revealed by measures of diurnal patterns of salivary cortisol compared to a more integrated measure of cortisol levels over time as evaluated in hair. Importantly, consistent with our hair cortisol findings in the present study, we found that the extent of pain-related stress adjusted for neonatal medical confounders was associated with reduced salivary cortisol levels on the test day, and salivary diurnal cortisol pattern on non-school days, primarily in boys (Grunau and colleagues unpublished data). Our findings that the relationship between neonatal pain-related stress and down regulation of cortisol at age 7 years converges across multiple independent cortisol measures speaks to the robustness of our findings.

NF-κB belongs to a family of transcription factors that can orchestrate many stress and inflammatory processes. Activation of NF-κB represents a downstream effector for the response to stressful events and links changes in the activity of the neuroendocrine axis to the cellular response [49]. An example of this link is the finding by Miller and colleagues demonstrating heightened expression of genes controlled by NF-κB in adults who experienced low socioeconomic status (SES) in early life [50]. Genetic variation in the promoter region of *NFKBIA*, which alters the ‘tuning’ of stress and immune responsiveness, has been linked to alterations in susceptibility to infectious and inflammatory diseases, and a variety of cancers [28], [51]. In this study we identified a novel association in boys between *NFKBIA* rs2233409 genotype, hair cortisol and neonatal procedural pain-related stress. Intriguingly, rs2233409 lies in the putative binding site of octamer binding transcription factor-1 (Oct-1) that interacts synergistically with the glucocorticoid receptor (GR) to bind DNA [52]. Disruption of Oct-1 binding due to the rs2233409 polymorphism would be predicted to reduce the anti-inflammatory effects of ligand bound GR, since DNA binding of Oct-1 strictly depends on GR binding [53]. Our present findings that the minor allele (CT or TT) of *NFKBIA* rs2233409 may influence the long-term effects of neonatal pain-related stress provide evidence of an association only, and animal experimental work is required to establish a causal pathway. We have shown that in boys born preterm, the minor allele (CT or TT) of *NFKBIA* rs2233409 was associated with higher secretion of inflammatory cytokines, providing support to the idea that neonatal pain-related stress may act as a proinflammatory stimulus that induces long-term immune cell activation. Further research is needed to elucidate the functional implications of this immune system reprogramming effect.

### Gender Differences in the Effects of Early Stress on HPA Axis Function

Importantly, boys with the minor allele (CT or TT) for *NFKBIA* rs2233409 displayed the greatest vulnerability to long-term effects of neonatal pain-related stress. In animal studies, early life stress can have sex-dependent long-term effects on the responsiveness of the HPA axis [54]–[56], with some studies reporting higher vulnerability or sensitivity in males to the early disturbances compared to females (e.g. [57], [58]), while others report greater vulnerability or sensitivity predominantly in females [59], [60]. These discrepancies may be due to different types and timing of the stressors [61] and suggest that males and females may either have different developmental windows of vulnerability and/or adapt differently to the early adverse environment.

Human longitudinal studies on gender differences in HPA axis programming due to early adverse experiences are rare, and to our knowledge this is the first to investigate the relationship of early pain-related stress to HPA axis function comparing boys and girls born very preterm. A previous study by Jones et al., [62] found that in 7–9 year old children, lower birth weight was significantly associated with a higher salivary cortisol response to the Trier Social Stress Test (TSST) in boys, but not in girls. In line with this, Elzinga et al. 2008 [63] reported a blunted cortisol response to the TSST in subjects with early trauma, which was more prominent in men than in women. However, the direction of effects can vary, and in some studies no gender differences were found in neuroendocrine regulation after early stress or trauma (for review see: [64]. Our findings extend these previous data, demonstrating downregulation of HPA activity in hair cortisol, which provides an integrated measure of cortisol levels over time.

Taken together, early life stress appears to be more strongly associated with HPA axis function in males than in females [64], consistent with our current results of a negative association of early life pain-related stress and hair cortisol in preterm boys but not girls. Importantly, similar differences in prepubertal children have been reported previously [62]. Thus variation in concurrent sex steroid concentrations cannot sufficiently explain the observed gender differences [64]. Instead, evidence from animal studies suggests that there may be an important interaction between early stress and regulation of gonadal hormone production, suggesting that early adverse experiences may interfere with gonadal programming of a sexually dimorphic brain [61]. However, little is known about disturbances of the normal hormonal milieu and surges due to premature birth and early stress exposure. It is clear that more research is needed to better understand the impact of gender on neuroendocrine function after early life stress in this population, and relationships between HPA axis regulation and clinical problems.

We have recently reported that infants born very preterm exposed prenatally to chorioamnionitis with funisitis (inflammation of the umbilical cord) had a different pattern of cortisol levels at 18 months corrected age, compared to infants exposed to chorioamnionitis alone or no prenatal infection, suggesting that presence of a *fetal* inflammatory response may alter programming of the HPA axis. In the present study, in school-age children, we did not have the results of placental histopathology, which places a limitation on our current findings. However, this does not alter our conclusion that functional genetic variation of stress and inflammatory responses appears to play a role in altered cortisol expression in boys born very preterm. Another important limitation to the present study is that the sample size was too low to evaluate the role of factors such as sources of current child stress (beyond parent depression and trait anxiety), socioeconomic status, parent-child behavioral interactions, and childhood illness after the NICU. In fact, given the wide range of past and concurrent factors that might affect the expression of cortisol, it was noteworthy that the association between neonatal pain-related stress and hair cortisol persisted to school-age (albeit only in boys).

## Conclusions

Altered programming of the HPA axis indexed by cumulative cortisol levels, persists to school age in children born very preterm. In contrast to the elevated cortisol levels observed previously at 8 and 18 months, at age 7 years, cortisol was downregulated in preterm relative to full-term children. These findings suggest that these children may be experiencing some level of chronic stress over the long term, consistent with the pattern seen in post-traumatic stress disorder long after the stressor has ended. Importantly, this study is the first, to our knowledge, to reveal that functional genetic variation in the promoter of *NFKBIA*, which is a critical negative regulator of stress and inflammatory processes, plays a key role in long-term programming effects of neonatal pain-related stress on HPA function in children born very preterm. Furthermore, higher secretion of inflammatory cytokines was related to the minor allele (CT or TT) of *NFKBIA* rs2233409, consistent with the possibility that neonatal pain-related stress may act as a proinflammatory stimulus that induces long-term immune cell activation. The finding that these effects were gender-specific, with boys driving the association, has major clinical implications for understanding possible gender differences in vulnerability of these children.
